# Correction to “Relationship between objective cognitive functioning and work performance among Japanese workers”

**DOI:** 10.1002/1348-9585.12416

**Published:** 2023-07-04

Shibaoka M, Masuda M, Iwasawa S, Ikezawa S, Eguchi H, Nakagome K. J Occup Health. Jan 2023;65(1):e12385. doi:10.1002/1348‐9585.12385

In Table 1, the category of educational years was incorrect. The correct numbers of educational years are as follows.

**TABLE 1:** Sociodemographic characteristics.

	*n* = 276	(%)	Mean	(SD)	High‐performing group (*n* = 187)				Low‐performing group (*n* = 89)			
					*n*	(%)	Mean	(SD)	*n*	(%)	Mean	(SD)
Age (years)			45.2	(10.2)			45.1	(10.1)			45.4	(10.4)
Sex												
Male	178	(64.5)			113	(60.4)			65	(73.0)		
Female	98	(35.5)			74	(39.6)			24	(27.0)		
Other	0	(0.0)			0	(0.0)			0	(0.0)		
Educational years												
≤12	41	(14.9)			29	(15.5)			12	(13.5)		
13–16	181	(65.6)			121	(64.7)			60	(67.4)		
≥17	54	(19.6)			37	(19.8)			17	(19.1)		
Marital status												
Married (including living with common‐law partner)	193	(69.9)			132	(70.6)			61	(68.5)		
Single (including divorced and widowed)	83	(30.1)			55	(29.4)			28	(31.5)		
Employment status												
Full‐time worker	230	(83.3)			154	(82.4)			76	(85.4)		
Part‐time worker	46	(16.7)			33	(17.6)			13	(14.6)		
Employment position												
Manager	77	(27.9)			53	(28.3)			24	(27.0)		
Nonmanager	199	(72.1)			134	(71.7)			65	(73.0)		
Sleep difficulties (AIS)												
≥6	117	(57.6)			59	(31.6)			58	(65.2)		
≤5	159	(68.4)			128	(68.4)			31	(34.8)		
Physical disease treatment status												
Under treatment	65	(23.6)			47	(25.1)			18	(20.2)		
Not treated	211	(76.4)			140	(74.9)			71	(79.8)		
Mental illness treatment status												
Under treatment	16	(5.8)			5	(2.7)			11	(12.4)		
Not treated	260	(94.2)			182	(97.3)			78	(87.6)		
Depression tendency (K6)												
≥5	124	(44.9)			65	(34.8)			30	(66.3)		
≤4	152	(55.1)			122	(65.2)			59	(33.7)		
ADHD tendency (ASRS v.1.1 part A)												
+	35	(12.7)			16	(8.6)			19	(21.3)		
−	241	(87.3)			171	(91.4)			70	(78.7)		
Autistic traits (AQ‐J)												
≥7	18	(6.5)			9	(4.8)			9	(10.1)		
≤6	258	(93.5)			178	(95.2)			80	(89.9)		
Resilience (RS‐14)			65.3	(14.7)			68.8	(12.5)			58.0	(16.4)
QOL (WHOQOL 26)			3.3	(0.6)			3.2	(0.8)			2.8	(0.8)
Cognitive functioning (THINC‐it®)												
Spotter (ms)			554.93	(135.8)			543.5	(123.6)			578.9	(156.6)
Symbol check (number)			28.717	(10.0)			29.4	(9.9)			27.3	(10.2)
Codebreaker (number)			60.812	(15.2)			60.7	(14.6)			61.2	(16.6)
Trails (s)			26	(11.7)			25.9	(10.9)			26.3	(13.4)
PDQ‐5D (point)			4.13	(2.9)			3.9	(2.7)			4.6	(3.2)
Spotter (ms)												
Low (344.73–485.62)	92	(33.3)			69	(36.9)			23	(25.8)		
Moderate (485.63–595.38)	91	(33.0)			62	(33.2)			29	(32.6)		
High (595.39–1490.00)	93	(33.7)			56	(29.9)			37	(41.6)		
Symbol check (number)												
Low (0–26)	95	(34.4)			61	(32.6)			34	(38.2)		
Moderate (27–35)	91	(33.0)			57	(30.5)			34	(38.2)		
High (36–40)	90	(32.6)			69	(36.9)			21	(23.6)		
Codebreaker (number)												
Low (3–56)	94	(34.1)			59	(31.6)			35	(39.3)		
Moderate (57–67)	91	(33.0)			70	(37.4)			21	(23.6)		
High (58–108)	91	(33.0)			58	(31.0)			33	(37.1)		
Trails (s)												
Low (11.05–20.47)	92	(33.3)			64	(34.2)			28	(31.5)		
Moderate (20.48–27.37)	92	(33.3)			61	(32.6)			31	(34.8)		
High (27.38–103.07)	92	(33.3)			62	(33.2)			30	(33.7)		
PDQ‐5D (points)												
Low (0–3)	123	(44.6)			86	(46.0)			37	(41.6)		
Moderate (4–5)	69	(25.0)			45	(24.1)			24	(27.0)		
High (5–15)	84	(30.4)			56	(29.9)			28	(31.5)		

*Note*: Spotter (Choice Reaction Time) = mean latency for correct response. Symbol check (N‐back) = total number of correct responses. Codebreaker (Digit Symbol Substitution Test) = total number of correct responses. Trails (Trail Making Test) = total time taken for completion. PDQ‐5D (Perceived Deficits Questionnaire for Depression‐5 item) = total score (points).

Abbreviations: ADHD, attention deficit hyperactivity disorder; AIS, Athens Insomnia Scale; AQ‐J, Autism‐Spectrum Quotient‐Japanese version; ASRS, Adult ADHD Self‐Report Scale v.1.1 part A; K6, Kessler Psychological Distress Scale; QOL, quality of life; SD, standard deviation.

We apologize for this error.

